# Neurological sequelae after encephalitis associated with herpes simplex virus in children: systematic review and meta-analysis

**DOI:** 10.1186/s12879-023-08007-3

**Published:** 2023-01-26

**Authors:** Natalie Duran Rocha, Sara Kvitko de Moura, Gabriel Aude Bueno da Silva, Rita Mattiello, Douglas Kazutoshi Sato

**Affiliations:** 1 Programa de Pós-Graduação em Pediatria e Saúde da Criança da PUCRS, Pontifícia Universidade Católica do Rio Grande Do Sul, Av. Ipiranga, 6681 – Bairro Partenon, Porto Alegre, RS 90619-900 Brazil; 2 Curso de Graduação em Medicina da Escola de Medicina da PUCRS – Pontifícia Universidade Católica do Rio Grande do Sul, Av. Ipiranga, 6681 – Bairro Partenon, Porto Alegre, RS 90619-900 Brazil; 3 Programa de Pós-Graduação em Epidemiologia Universidade Federal do Rio Grande do Sul, Av. Paulo Gama, n° 110 –Bairro Farroupilha,, Porto Alegre, RS 90040-060 Brazil

**Keywords:** Encephalitis, Herpes simplex virus, Neurological manifestations, Children and adolescents

## Abstract

**Background:**

Encephalitis is an inflammation of the cerebral parenchyma manifested by acute symptoms such as fever, headaches, and other neurological disorders. Its etiology is mostly viral, with herpes simplex virus being a frequent etiological agent in children. The development of neurological sequelae is a serious outcome associated with this infection.

**Objective:**

To assess the general prevalence and types of neurological sequelae in children after a case of acute viral encephalitis caused by HSV.

**Methods:**

This systematic review and meta-analysis was developed following the PRISMA guidelines. The literature search was carried out in the MEDLINE, Embase, SciELO, LILACS, Cochrane, CINAHL, PsycINFO, and Web of Science databases. Studies were included of children with confirmed HSV infection and that presented a description of neurological sequelae associated with that infection. For the meta-analysis of general prevalence and of the types of neurological sequelae a random effects model was used.

**Results:**

Of the 2827 articles chosen in the initial search, nine studies were included in the systematic review and meta-analysis. The general prevalence of neurological sequelae was 50.7% (95% CI 39.2–62.2). The most frequent sequelae were related to mental disability, with a 42.1% prevalence (95% CI 30–55.2); on the other hand, the least frequent sequelae were those related with visual impairment, with a 5.9% prevalence (95% CI 2.2–14.6). The included studies presented regular quality and substantial heterogeneity.

**Conclusion:**

Even with antiviral therapy, half of patients will develop some type of disability.

**Supplementary Information:**

The online version contains supplementary material available at 10.1186/s12879-023-08007-3.

## Background

Encephalitis is an inflammation of the cerebral parenchyma characterized by clinical manifestations associated with neurological dysfunction [1]. Signs or symptoms such as an altered mental state, fever, and neurological deficits, which are presented acutely, should be investigated for a possible encephalitis diagnosis [2].

The etiology can be infectious or non-infectious, with viruses being responsible for the greatest number of cases when an infectious cause is identified [3]. Among these viruses, herpes simplex virus (HSV) is responsible for most cases in pre-school children [2]. The test of choice for confirming the diagnosis is usually viral DNA (deoxyribonucleic acid) detection by PCR (polymerase chain reaction) in cerebrospinal fluid (CSF) [4]. After clinical suspicion of encephalitis, the recommendation is to begin acyclovir treatment as early as possible, in order to avoid possible neurological damage associated with inflammation of the cerebral parenchyma [5].

The consequences of acute viral encephalitis caused by HSV can lead to high morbimortality rates. Mortality can reach 70% when untreated and it is estimated that even with administration of the recommended therapy after the onset of the disease almost two-thirds of patients can go on to die or will present expressive and permanent residual neurological deficits [6, 7]. A systematic review published in 2016 showed that 42% of children presented an incomplete recovery in the follow-up after infection, that is, they manifested some type of neurological sequela [7]. That same study contains a subanalysis that showed 64% prevalence of children with some type of neurological sequelae after HSV encephalitis. However, it is important to highlight that this study includes different etiological agents as well as a broad age group, with patients in the neonatal period.

Although neurological sequelae are a major consequence of HSV encephalitis in children, the degree of that consequence is unknown. This shows the importance of us understanding the prevalence of neurological sequelae after HSV encephalitis, so that we can in some way identify how much we can improve the outcome and, consequently, the prognosis of this devastating disease with such impactful damage to the children’s future. Therefore, the aim of this systematic review was to assess the general prevalence and types of neurological sequelae in children after a case of acute viral encephalitis caused by HSV.

## Methods

### Protocol and registration

This systematic review was carried out according to the Preferred Reporting Items for Systematic Reviews and Meta-Analyses (PRISMA) guidelines [8] and the protocol was registered in the International Prospective Register for Systematic Reviews (PROSPERO), under registration number CRD42021225536.

### PEO strategy

The PEO strategy was defined as follows: “P”: children and adolescents between 2 months and 18 years old; “E”: HSV infection; and “O”: neurological sequelae after a case of acute viral encephalitis.

### Inclusion criteria

We included observational studies of children and adolescents between 2 months and 18 years old who had an HSV infection proven by laboratory test and for whom the studies described data about neurological sequelae after acute viral encephalitis.

### Exclusion criteria

We excluded review articles, cases series, studies with fewer than 10 patients with HSV infection, editorials, and letters to editors. Articles published before 1980 were also excluded, since in that period acyclovir treatment was not yet established [6, 9].

### Information sources and search

The databases used to search the literature were the following: MEDLINE (via PubMed), Excerpta Medica (Embase), The Cochrane Central Register of Controlled Trials (CENTRAL), Scientific Electronic Library Online (SCIELO), Latin American Caribbean Health Sciences Literature (LILACS via BIREME), Cumulative Index to Nursing and Allied Health Literature (CINAHL), Web of Science, and American Psychological Association PsycINFO. We also searched in the gray literature and in the reference lists of the selected articles. The MEDLINE search strategy was created and adapted for the other databases. The search was conducted in December of 2021 and did not include any language restrictions (Additional file 1).

### Study selection

Two researchers (NDR and SKM) carried out the study selection independently. A third reviewer (ALT) verified the contentious articles in order to reach a consensus. The selection process occurred in two stages. The first stage consisted of the study selection based on reading the titles and abstracts. In the second stage, the articles selected in the first stage were read in full. Cohen's kappa test was used for the agreement analysis. The cut-off point considered was kappa ≥ 0.8 for ideal agreement [10].

### Data extraction

The information on the chosen works was summarized in a standard form, organized so as to extract the data considered to be relevant from each one of the primary studies. The objective was to structure the data and guarantee consistency in the extraction process. The information extracted from each study was: general information (author, year of publication, country); characteristics of the studies (design, inclusion and exclusion criteria); study population (total number of patients with HSV infection, mean age, sex, diagnostic method for identifying HSV, initial symptoms of acute viral encephalitis, treatment type, dose and duration, outcome assessment time, total patients with neurological sequelae, and types of disabilities). In the data extracted for the systematic review, the neurological symptoms, type of sequelae, and disability were presented according to the original articles. However, for the metanalysis, due to their heterogeneity of then and their low frequency, we created categories associating the clinically closest characteristics.

For the information that was not described in the articles, the authors were contacted (at least three times, in different periods, more than 4 weeks apart) via email.

### Assessment of study quality and risk of bias

The quality of the primary studies was assessed independently by two authors of the review (NDR and SKM) according to the National Institute of Health Study Quality Assessment Tools for cohort and cross-sectional studies [11]. This tool contains 14 items (questions) and was developed to assess the internal validity of studies, analyzing faults in the design or implementation. Each item can be judged with “yes” (study fulfills that item), “no” (study does not fulfill that item), or “other” (impossible to determine, not applicable, or not reported). The final classification is made after analyzing the answers to these 14 items, with the final result being either “good,” “average,” or “bad.” The main objective of this tool is to assess the strength of causal association between an exposure variable and an outcome variable.

### Data analysis

To analyze the prevalence of neurological sequelae we considered as a denominator the total number of patients with HSV encephalitis reported in the studies. To analyze the prevalence of the types of neurological sequelae, we considered as a denominator the total number of neurological sequelae described.

For the meta-analysis we calculated the general prevalence (95% confidence interval [CI]) of neurological sequelae and of the types of neurological sequelae of each study. Considering the possibly high heterogeneity among the studies, the prevalence rates were estimated using random models. The heterogeneity of the studies was assessed using the I^2^ statistical technique; this method is a percentage measure of heterogeneity that ranges from 0% (no heterogeneity) to 100% (total heterogeneity). I^2^ values < 40% were considered as unimportant heterogeneity; I^2^ between 40 and 60% as moderate heterogeneity; 60% to 90% as substantial; and between 90 and 100% as considerable heterogeneity [12]. Associations with p < 0.05 were considered statistically significant.

To analyze the general prevalence of neurological sequelae according to the classification of countries we considered the HDI (Human Development Index) relating to the year of publication of the study. The HDI is divided into four levels: very high human development (HDI between 0.800 and 1), high human development (HDI between 0.700 and 0.799), average human development (HDI between 0.550 and 0.699), and low human development (HDI below 0.549). In our study, we considered countries with a HDI ≥ 0.800 as developed, countries with a HDI between 0.799 and 0.550 as developing, and countries with a HDI < 0.549 as undeveloped [13].

To describe the symptoms at the onset of encephalitis we considered any of the following symptoms reported in the primary studies as a change of mental state suggested by encephalopathy: disorientation, change in consciousness, change of mental state, drowsiness, and coma.

The types of neurological sequelae described in the studies were classified into five major groups: **seizures **(convulsions, focal or generalized epilepsy, paroxistic tonic–clonic movements with or without loss of consciousness)** motor disabilities** (low muscle tone, abnormal movement pattern, tetra/hemiparesis, motor skills disorders, motor delay, limb paralysis, focal deficit, hemiplegia, ataxia), **visual impairments** (reduced visual acuity, visual sequelae, eye movement disorders), **mental disabilities** (global development delay, aphasia, dysarthria, altered speech, and other language disturbances, compromised short and/or long-term memory loss, cognitive dysfunction, executive dysfunction, psychiatric symptoms, global development delay, irritability, trouble holding attention on tasks, emotional disorders, altered speech, altered mental state, encephalopathy, cognitive dysfunction or delay, change of personality, behavioral sequelae, autism), and **others** (areflexia, hyperreflexia, sensory disturbances, cranial nerve palsy).

### Ethics

According to the rules of Resolution 466/12 of the National Health Council [14], the project is classified as a systematic review of information in secondary sources, with no personal identification of patients. For that reason, the assessment and approval of the ethics committee is not needed. However, the present study was assessed and approved by the Scientific Commission of the School of Medicine of the Catholic Pontifical University of Rio Grande do Sul (PUCRS), besides being registered in the PROSPERO platform.

## Results

**Fig. 1:**
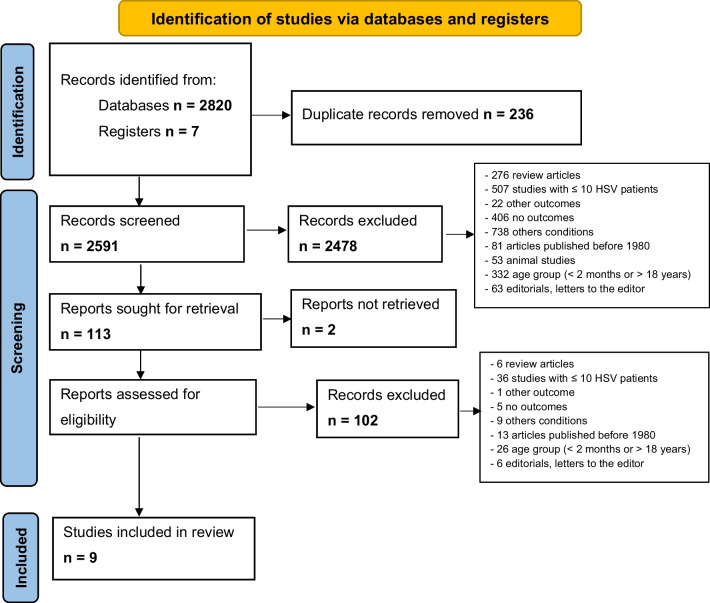
Study search per the PRISMA guidelines (the figure was produced using Word 2010)

The search identified 2827 articles in the included databases and in the gray literature. After excluding the duplicates (236), 2591 studies were screened by reading the titles and abstracts. In this stage, 2478 articles were excluded. Of the 113 articles chosen to read in full, nine were included in the present review. We did not obtain any answer from the authors contacted in relation to the data that were missing in the included studies. In the first stage of screening, the kappa agreement coefficient between the reviewers was 0.84; in the second stage, it was 0.89. The flowchart of the study selection is illustrated in Fig. 1.

### Characteristics of the studies

The included articles were published between 1996 [15] and 2018 [16, 17] and are from eight different countries, including articles from Canada [18], Great Britain [19], Germany [20], Poland [15], India [17], Israel [21], Japan [22], and China [16, 23]. According to the year of publication of the articles, five originated from developed countries [18–22] and four from developing countries [15–17]. Eight studies had a cross-sectional design (89%) [15–21, 23] and one study had a case–control design [22]. The population of the studies included children from 2 months [18] to 16 years old [20–22] from hospitals in the region where the study was conducted. The eligibility criteria for the studies varied; five of them included children with HSV encephalitis confirmed by the presence of that etiological agent in fluid [15, 20–23], and another four included children with neurological diseases (encephalopathy, meningitis, or encephalitis) but without the need to identify the etiological agent for inclusion in the study [16–19]. Of the latter, all sought the etiological agent responsible for the neurological alterations, with our systematic review only considering the sample of patients with confirmation of the presence of HSV. The exclusion criteria were described in four studies [16–18, 22]; two studies excluded neonatal patients [16, 22] and three studies excluded patients with another neurological disease [16–18]. The characteristics of the studies are presented in Table 1.

**Table 1:** Characteristics of the included studies

First author, year	Country HDI	Type of study	Eligibility criteria	Total HSV patients	Mean, age years (min–max)	Sex F/M	HSV diagnosis, n (%)	Symptoms at onset, n (%)	Type of treatment (%) Dose Duration days (min–max)	Time to follow-up	Sequelae, n (%)
Schleede L, 2012	Germany 0.934	Cross-sectional	Inclusion criterion: Proven herpes simplex virus encephalitis as shown by a positive polymerase chain reaction in cerebrospinal fluid or serologic evidence such as a significant rise in serum IgG-anti herpes simplex virus antibodies combined with a positive antibody-specific index Exclusion criteria:?	32	7.5 (5 months–16 years)	9/23	Liquor PCR, 31 (97%)	Fever, 20 (63%) Headache, 14 (44%) Fatigue, 11 (34%) Vomiting, 10 (31%) Dizziness, 4 (13%) Diarrhea, 2 (6%) Abdominal pain, 1 (3%) Neurological symptoms, 12 (38%) Seizures,7 (58%) Meningism, 3 (25%) Disorientation, 3 (25%)	Acyclovir (100%) Dose: 40 mg/kg/d (20–60 mg/kg/d) Duration (10–36)	Discharge from hospital	18 (56.2%)
Elbers JM, 2007	Canada 0.896	Cross-sectional	Inclusion criteria: Encephalopathy, defined as depressed or altered level of consciousness persisting for > 24 h, plus ≥ 2 of the following: fever (> 38 °C), seizure, focal central nervous system (CNS) findings, CSF pleocytosis (> 5 × 106 cells per L), EEG abnormalities, or diagnostic imaging abnormalities (in brain computed tomography [CT]/MRI scans) Exclusion criteria: Patients who had an underlying neurologic disease or were known to have immunosuppression	16	5.5 (2 months–14 years)	8/8	Liquor PCR, 12 (75%)	Fever, 16 (100%) Focal seizure, 11 (69%) Hemiparesis, 5 (31%) Dysphasia, 2 (13%)	Acyclovir (100%) Dose? Duration (7–21)	Minimum: 3 months after discharge Mean: 4 years	10 (62.5%)
Yoshinori I, 1998	Japan 0.849	Case–Control	Inclusion criteria: Patients aged 3 months to 16 years in whom HSE was diagnosed between September 1982 and January 1995. The diagnosis of HSE was confirmed by the detection of HSV DNA in CSF by the PCR assay. They also studied patients aged 1 month to 15 years with other infections of the central nervous system. In all control subjects the CSF in the acute phase was negative for HSV DNA by PCR Exclusion criteria: Neonates with HSV	24	5 (3 months–16 years)	8/16	Liquor PCR, 24 (100%)	Fever, 24 (100%) Seizures, 22 (92%) Initial neurological symptoms Seizures (9/20): 45% Altered consciousness (7/20): 35% Dysarthria (3/20): 15% Glasgow on admission: ≥ 11 (2/23) 9% 7–10 (8/23) 35% ≤ 6 (13/23) 56%	Acyclovir (100%) Dose 30 mg/kg/day Duration (10–14)	6 months	17 (70.8%)
Chen T, 2018	China 0.758	Cross-sectional	Inclusion criteria: Patients who had a clinical diagnosis of acute CNS infection. Patients with meningitis and/or encephalitis was defined as those with (1) an acute onset of illness; (2) at least one abnormality of the cerebrospinal fluid (CSF): WBC count > 15 cells/mm^3^ or protein level > 400 g/L; (3) temperature > 38 °C; and (4) decreased consciousness, seizures, altered mental status or focal neurological signs Exclusion criteria: (1) aged ≤ 28 days or aged ≥ 16 years; (2) tuberculous meningitis; (3) proven or probable noninfectious etiology of CNS disease	15	1.9 (4 months–7.7 years)	?	Viral antibody IgM and/or DNA/RNA in CSF	?	Acyclovir (100%) Dose 30 mg/kg/d Duration (14–24)	3.8–4.7 years after discharge	10 (66.6%)
Kumar R, 2018	India 0.647	Cross-sectional	Inclusion criteria: Patients from 1 mo to 16 y with acute encephalitis syndrome were included in the study. The classification of AES was based on the World Health Organization (WHO) Exclusion criteria: Patients with non-viral etiology (acute bacterial meningitis, tubercular meningitis, cerebral malaria, and electrolyte imbalance)	23	4.2	1:1.9	Serology, culture and/or PCR (liquor)	Fever, 23 (100%) Change in mental status, 23 (100%)	?	Discharge from hospital	7 (30.4%)
Zhang R, 2016	China 0.746	Cross-sectional	Inclusion criteria: Patients were diagnosed as having encephalitis and also had positive PCR for HSV from cerebrospinal fluid, and/or positive immunoglobulin M (IgM), from July 1996 to June 2007 in the Pediatric Department of Qingdao Municipal Hospital Exclusion criteria:?	36	6.43 (9 months–15 years)	15/21	Liquor PCR and/or HSV-IgM	Fever, 28 (78%) Seizures, 17 (47%) Somnolence, 15 (42%) Coma, 12 (33%) Vomiting, 12 (33%) Behavioral change, 5 (14%) Headache, 3 (8%)	Acyclovir (72%) Dose? Duration?	?	13 (36%)
Lahat E, 1999	Israel 0.859	Cross-sectional	Inclusion criteria: Patients aged between 9 months and 16 years with a diagnosis of HSV, who were treated in the pediatric department of the two university affiliated medical centers near Tel Aviv, Israel, from January 1984 until June 1995 Exclusion criteria?	28	7.16 (9 months–16 years)	6/22	Antibody anti-HSV (liquor) HSV IgM and IgG (ELISA) PCR (liquor)	Fever, 22 (79%) Altered consciousness, 19 (68%) Personality changes, 12 (43%) Headache, 14 (50%) Vomiting, 16 (57%) Seizures, 19 (68%)	Acyclovir (100%) Dose: 30 mg/kg/d Duration (8–10)	First years of follow-up to 5 years after HSV	10 (35.7%)
Woźniakowska-Gesicka T, 1996	Poland 0.753	Cross-sectional	Inclusion criteria: Children treated at the Department of Observation and Isolation and at the Department of Child Neurology at the Medical University of Warsaw in the years 1992–1994 with HSV. The diagnosis of HSV neuroinfection was made on the basis of changes in the cerebrospinal fluid in the form of lymphocytic inflammation and flies serological conditions Exclusion criteria:?	24	? (4 years–15 years)	9/15	HSV IgM antibody in CSF, 2 IgM anti-HSV antibody in the blood, 9 Increase IgG titers, 15	Fever, 16 (66%) Headache, 16 (66%) Neurological symptoms, 6 (25%) Vomiting, 5 (20,8%)	Acyclovir (100%) Dose 30 mg/kg/d Duration (mean 10)	6 months	8 (33.3%)
Ward KN, 2011	Britain and Ireland 0.906	Cross-sectional	Inclusion criteria: Children 2–35 months old with serious neurological disease between October 1998 and September 2001 according to the British Paediatric Surveillance Unit Exclusion criteria:?	19	0.97 (3 months–35 months)	13/6	HSV DNA detected in CSF or an HSV-specific intrathecal antibody response OR Seroconversion to IgG or IgM, within 2 weeks of onset of serious neurological disease	Fever, 18 (94%) Seizures, 19 (100%)—partial 12 (63%)	?	10 months–4 years (mean 2.3 years)	14 (73.6%)

HDI: Human Development Index; HSV: Herpes simplex virus; F: female; M: male; PCR: polymerase chain reaction; CNS: central nervous system; CSF: cerebrospinal fluid; EEG: electroencephalogram; CT: computed tomography; MR: nuclear magnetic resonance; HSE: Herpes simplex encephalitis; DNA: deoxyribonucleic acid; RNA: ribonucleic acid; WBC: white blood cells; WHO: World Health Organization; IgM: immunoglobulin M; IgG: immunoglobulin G

A total of 217 children with HSV infection were included in the systematic review. The mean age varied from 11 months [19] to 7.5 years old [20] and the predominant sex was the male sex [15, 20–23]. Regarding the etiological diagnosis of HSV encephalitis, the techniques used for viral identification were the presence of viral DNA using the PCR technique [17, 18, 20–23] and/or HSV IgM and IgG in fluid [15, 16, 19, 21, 23].

In relation to the symptoms at the onset of encephalitis, of the sample of 217 children, 167 (77%) presented fever [15, 17–23], 95 (43%) presented some type of convulsion [18–23], 79 (36%) presented an altered mental state [17, 20–23], 47 (21%) presented headaches [15, 20, 21, 23], and 43 (19%) presented vomiting [15, 20, 21, 23]. Other symptoms that also appeared at the onset of encephalitis but with a lower prevalence in the studies were fatigue (n = 11; 5%) [20], dizziness (n = 4; 2%) [20], diarrhea (n = 2; 1%) [20], abdominal pain (n = 1, 0.5%) [20], hemiparesis (n = 5, 2.3%) [18], dysphasia (n = 2, 1%) [18], dysarthria (n = 3; 1.4%) [22], and a change in behavior or personality (n = 17; 8%) [21, 23]. For the treatment of HSV encephalitis, acyclovir was prescribed in seven of the nine studies included [15, 16, 18, 20–23], and two studies did not specify in the text what treatment was used [17, 19]. In total, five studies described the acyclovir dose [15, 16, 20–22] and six studies described the duration of treatment [15, 16, 18, 20–22]. The dose prescribed in 80% of the studies was 30 mg/kg/day [15, 16, 21, 22] and the treatment time varied from 7 to 36 days [18, 20].

Most of the studies (77%) described the neurological sequelae developed by the patients [15, 16, 18–21, 24] and two studies did not describe them and their data were only used to assess the general prevalence of neurological sequelae [22, 23]. The moment of the outcome assessment varied from immediately on hospital discharge [17, 20] to 5 years after discharge [21]. Of the 217 patients included in the study, 107 (49.3%) presented neurological sequelae and 11 (5%) went on to die. The sum of all the sequelae described was 153. The types of neurological sequelae and impairments are described in Table 2.

**Table 2:** Neurological sequelae

First author, year	Total HSV patients	Patients with neurological sequelae, n (%)	Types of neurological sequelae, n	Type of disability, n (%)	Deaths
Schleede L, 2012	32	18 (56.2%)	Abnormal movement patterns, 8 Cranial nerve palsies, 6 Tetra/hemiparesis, 6 Aphasia, 4 Memory impairment, 2 Psychiatric symptoms, 2 Hyperreflexia, 2 Seizures, 1 Eye movement disorder, 1 Low muscle tone, 1 Sensory disturbance, 1 Arreflexia, 1	With disability: 35 Seizures: 1 (2.8%) Motor disability: 15 (42.8%) Visual impairment: 1 (2.8%) Mental disability: 8 (22.8%) Others: 10 (28.5%)	?
Elbers JM, 2007	16	10 (62.5%)	Seizures, 7 Global developmental delays, 4 Residual hemiplegia, 2	With disability: 13 Seizures: 7 (53.8%) Motor disability: 2 (15.3%) Visual impairment: 0 Mental disability: 4 (30.7%) Others: 0	?
Yoshinori I, 1998	24	17 (70.8%)	Mildly disabled (able to perform everyday activities but hampered by neurological defects), 3 Moderately disabled (does not require supportive care, but neurological defects influence daily life), 6 Severely disabled (requires supportive care), 8	With disability: 17 Seizure: ? Motor disability: ? Visual impairment: ? Mental disability: ? Others: ?	2
Chen T, 2018	15	10 (66.6%)	Irritability, 2 Trouble concentrating 5, Memory impairment, 4 Limb paralysis, 3 Ataxia, 3 Speech disorders, 4 Seizures, 4	With disability: 25 Seizures: 4 (16%) Motor disability: 6 (24%) Visual impairment: 0 Mental disability: 15 (60%) Others: 0	2
Kumar R, 2018	23	7 (30.4%)	Seizures, 5 Focal deficit, 1 Altered mental status, 3	With disability: 9 Seizures: 5 (55.5%) Motor disability: 1 (11%) Visual impairment: 0 Mental disability: 3 (33.3%) Others: 0	5
Zhang R, 2016	36	13 (36%)	Mild disability (neurological deficit does not affect normal life), 1 Moderate disability (neurological defects affect normal life, but do not require care), 11 Severe disability (cannot take care of themselves, require care), 1	With disability: 13 Seizure:? Motor disability:? Visual impairment:? Mental disability:? Others:?	0
Lahat E, 1999	28	10 (35.7%)	Cognitive dysfunction, 4 Personality change, 4 Speech abnormalities, 2 Motor skill disturbance, 5 Seizures, 4	With disability 19 Seizures: 4 (21%) Motor disability: 5 (26.3%) Visual impairment: 0 Mental disability: 10 (52.6%) Others: 0	2
Woźniakowska-Gesicka T, 1996	24	8 (33.3%)	Seizures, 3 Quadriparesis spastic, 1 Encephalopathy and emotional disorders, 4	With disability: 12 Seizures: 3 (25%) Motor disability: 1 (8.3%) Visual impairment: 0 Mental disability: 8 (66.6%) Others: 0	?
Ward KN, 2011	19	14 (73.6%)	Developmental cognitive and motor delay, 9 Developmental cognitive delay, 2 Motor system sequelae, 7 Behavioral sequelae, 1 Seizures, 7 Visual sequelae, 3 Autism, 1 Speech alterations, 1	With disability: 40 Seizures: 7 (17.5%) Motor disability: 16 (40%) Visual impairment: 3 (7.5%) Mental disability: 14 (35%) Others: 0	?

HSV: Herpes simplex virus

### Analysis of the quality of the studies

Following the classification of the National Institute of Health Study Quality Assessment Tools, six studies (66.6%) were classified as regular quality [15–18, 20, 21] and three studies (33%) were classified as low quality [19, 22, 23] (Table 3).

**Table 3:** Quality assessment of included studies

Primer author, year	1	2	3	4	5	6	7	8	9	10	11	12	13	14	Classification
Schleede L, 2012	Y	N	Y	Y	N	N	N	O	Y	O	N	O	O	N	Fair
Elbers JM, 2007	Y	Y	N	Y	N	N	N	O	Y	O	N	O	O	Y	Fair
Yoshinori I, 1998	N	N	N	O	N	N	N	O	Y	O	N	O	O	Y	Poor
Chen T, 2018	Y	Y	N	Y	N	N	N	O	Y	O	N	O	O	Y	Fair
Kumar R, 2018	N	Y	N	Y	N	N	N	O	Y	O	N	O	O	N	Fair
Zhang R, 2016	N	N	Y	Y	N	N	N	O	Y	O	N	O	O	Y	Poor
Lahat E, 1999	N	Y	Y	Y	N	N	N	O	Y	O	Y	O	O	N	Fair
Woźniakowska-Gesicka T, 1996	N	Y	Y	Y	N	N	N	O	Y	O	N	O	O	N	Fair
Ward KN, 2011	N	N	N	NR	N	N	N	O	Y	O	N	O	O	N	Poor

Y: yes; N: no; O: other (impossible to determine, not applicable, or not reported)

### Meta-analysis of the general prevalence and of the types of neurological sequelae

**Fig. 2:**
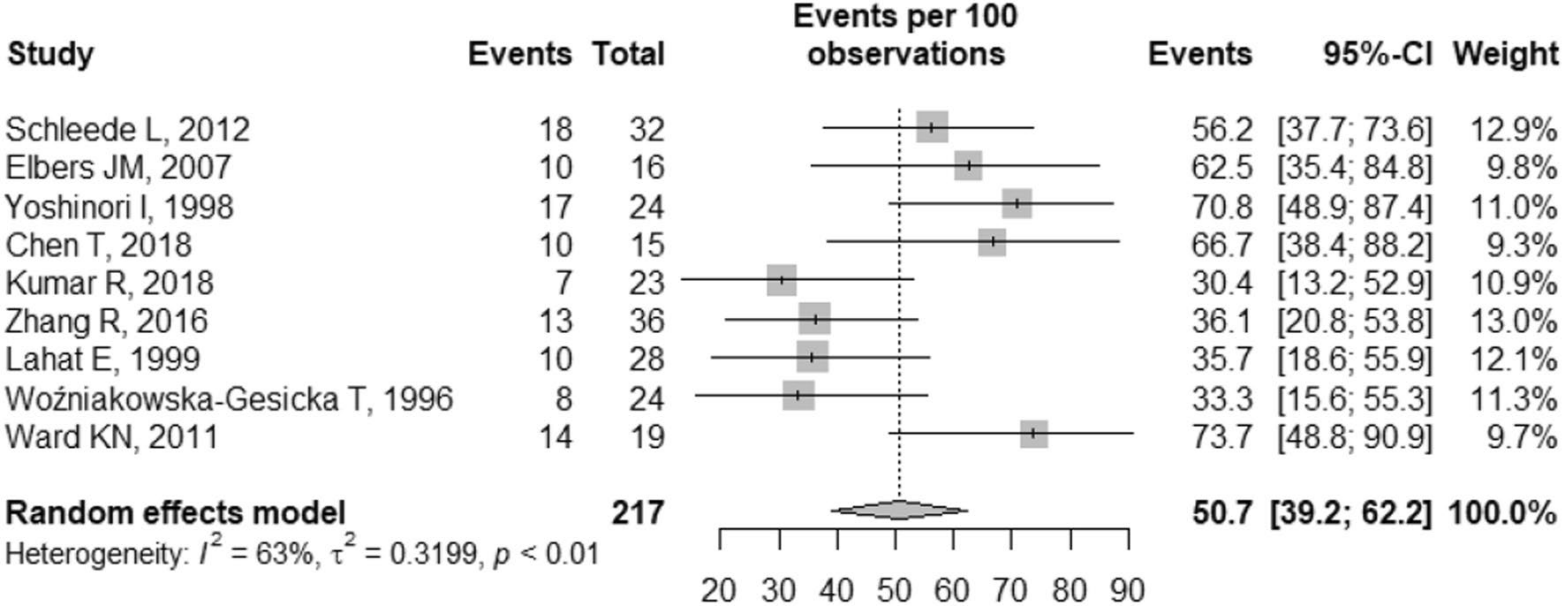
Forest plot of general prevalence of neurological sequelae. CI: confidence interval

**Fig. 3:**
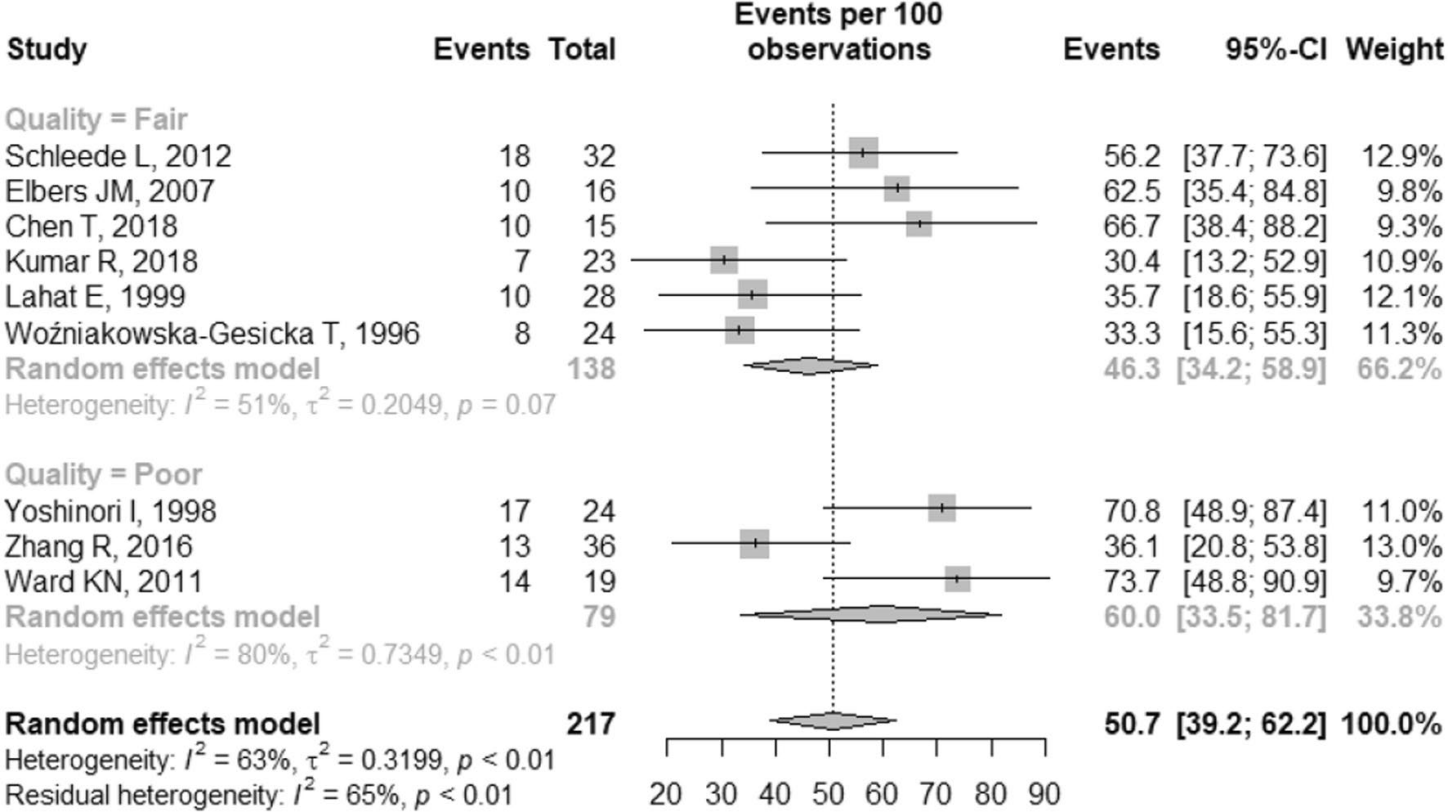
Forest plot of general prevalence of neurological sequelae according to the quality of the studies. CI: confidence interval

**Fig. 4:**
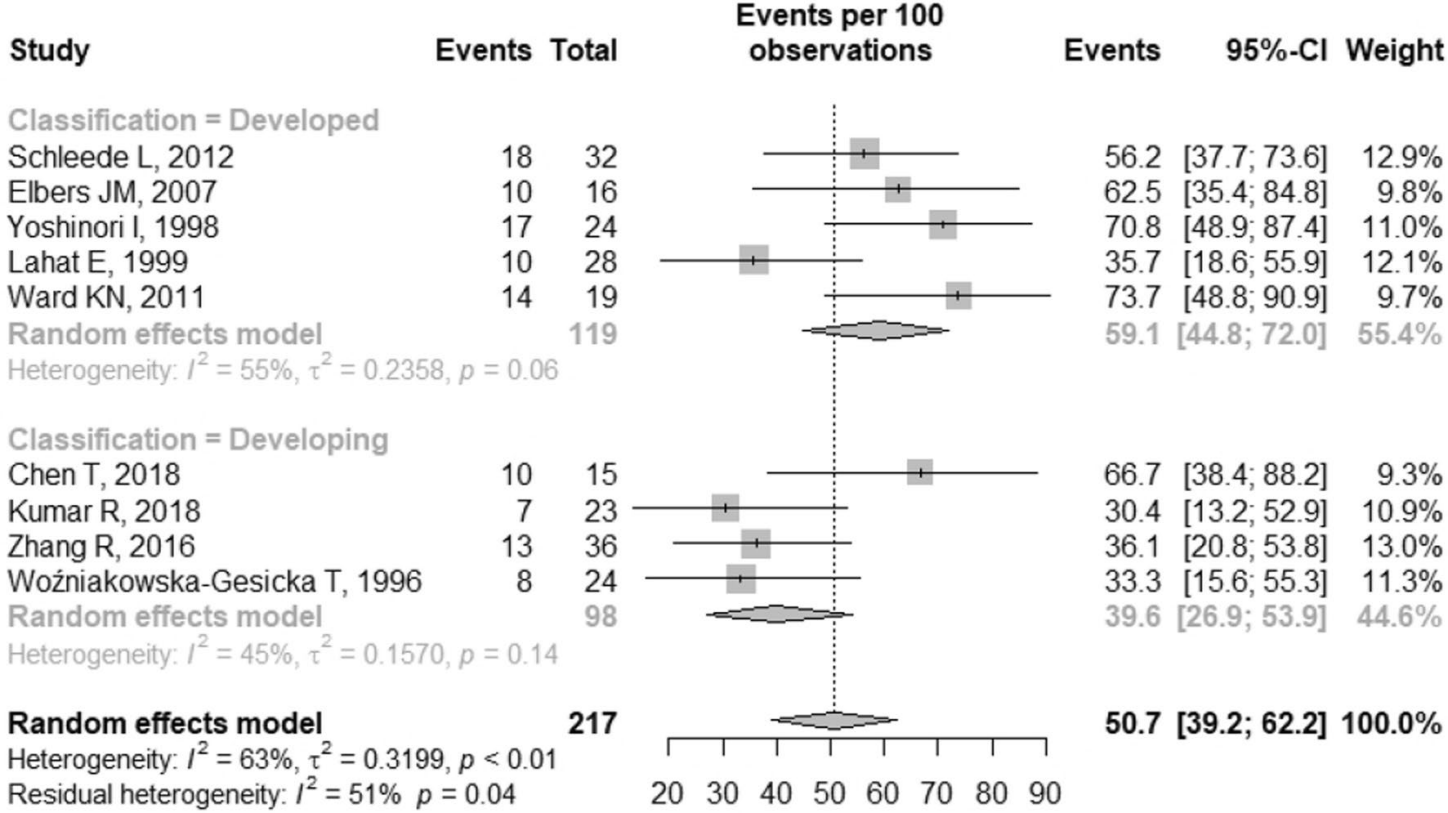
Forest plot of general prevalence of neurological sequelae according to the HDI of the countries. CI: confidence interval

The general prevalence of neurological sequelae was 50.7% (95% CI 39.2–62.2), I^2^ = 63% (Fig. 2). There was no statistically significant difference in the general prevalence result in relation to the quality of the studies (p = 0.369) (Fig. 3) and in relation to the classification of the countries according to the HDI (p = 0.05) (Fig. 4).

**Fig. 5:**
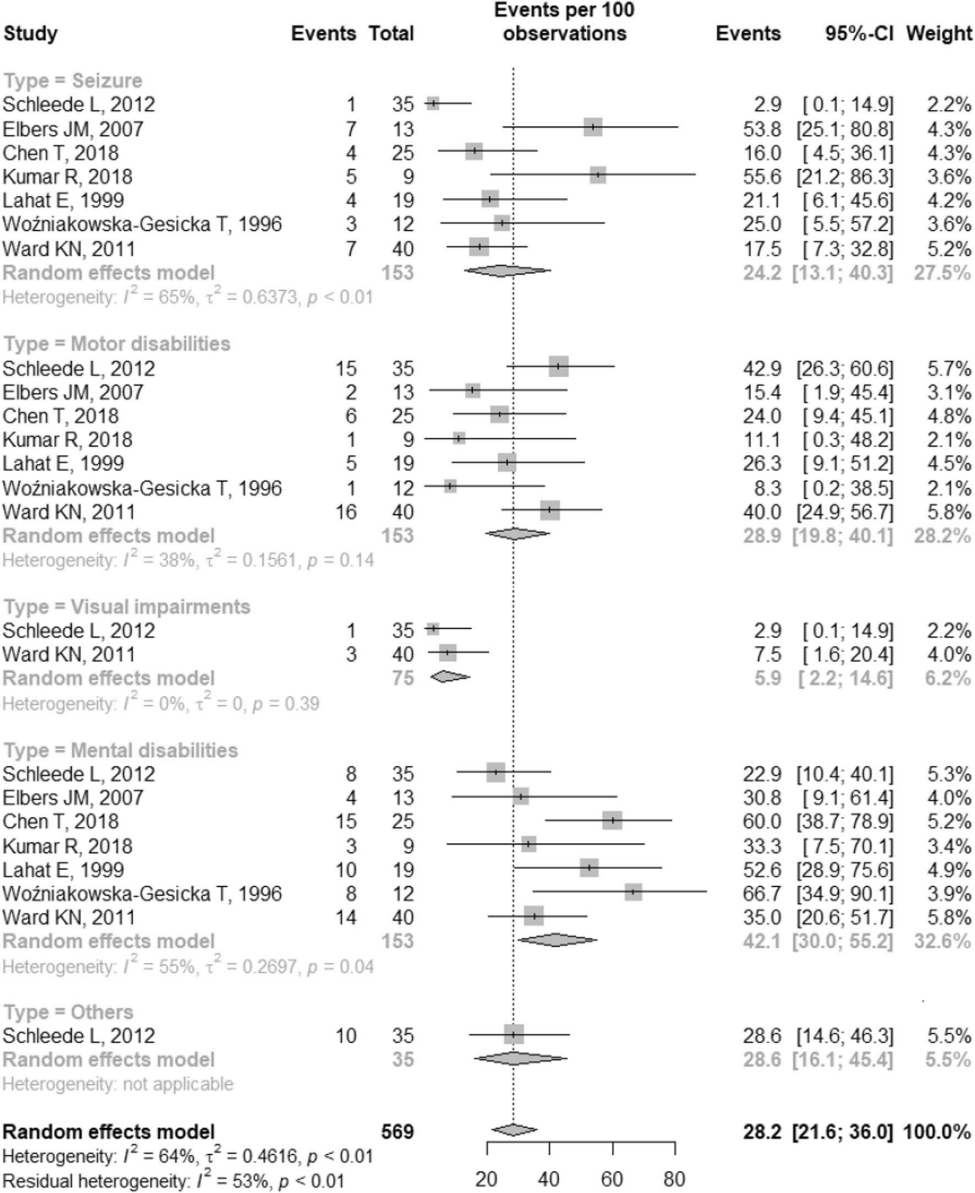
Forest plot of prevalence by type of disability. CI: confidence interval

The prevalence rates by types of impairment were: convulsion: 24% (95% CI 13.1–40.3), I^2^ = 65%; motor disabilities: 28.9% (95% CI 19.8–40.1), I^2^ = 38%; visual impairment: 5.9% (95% CI 2.2–14.6), I^2^ = 0%; mental disability: 42.1% (95% CI 30–55.2), I^2^ = 55%; and others 28.6% (95% CI 16.1–45.4), I^2^ = not applicable (Fig. 5).

## Discussion

As far as we know, this is the first systematic review and meta-analysis that describes the prevalence of neurological sequelae of encephalitis caused by HSV in children and adolescents. Half of the children with HSV encephalitis presented some type of neurological sequela, with mental disability being the most prevalent, and visual impairment being the least frequently described.

One possible explanation for the high prevalence of children with HSV encephalitis presenting neurological sequelae is the pattern of involvement of the virus. HSV results in acute inflammation, congestion, and/or bleeding, most prominently in the temporal lobes and adjacent limbic areas [25]. Over approximately 2 weeks, these lesions can evolve with greater involvement of the cerebral tissue, leading to irreversible damage to the CNS [25]. Besides these sequelae, cases of encephalitis associated with *N*-methyl-d-aspartate antibodies were more recently described in patients with previous HSV encephalitis, probably caused by an immune-mediated disease after massive exposure of secondary CNS antigens to the viral lesion [26]. This suggests the high ability of HSV to cause a brain lesion, both directly and indirectly.

Another hypothesis for this high prevalence is the duration of the treatment found. Some studies demonstrate that shorter courses of treatment may be associated with viral reactivation, with consequent persistence or intensification of the brain damage [27, 28]. We perceived that in our sample of patients, the treatment varied from 7 to 36 days; however, five studies referred to a minimum time far below that recommended [15, 18, 20–22] and only one study referred to a minimum time of 14 days [16]. As data were missing in the primary studies referring to the duration of treatment, it was not possible to analyze the relationship between treatment time and the development of neurological sequelae. However, it is valid to highlight that the guidance is to maintain treatment for 14 to 21 days. Shorter treatments could be considered incomplete and lead to the development of neurological sequelae.

The general prevalence of neurological sequelae found in our study is lower than the result described in the systematic review and meta-analysis published in 2016 regarding infectious encephalitis in children, which in one of its subanalyses showed 64% prevalence (95% CI 34.0–89.0%) of neurological sequelae in children with HSV encephalitis [7]. One possible explanation for that difference is that of the four studies included in that meta-analysis, one included children under 2 months and one was about the sequelae after encephalitis not exclusively associated with HSV. These characteristics may have changed the prevalence findings in that previous study compared with our study.

Most of the studies included in our final analysis are derived from developed countries according to the HDI classification; while a smaller portion originates from developing countries. There was no study published in an underdeveloped country. Despite us not finding any statistically significant difference in the general prevalence of neurological sequelae in developed countries and developing countries, we perceived the tendency for a greater prevalence of neurological sequelae in developed countries. This may be the result of greater monitoring of patients after acute diseases in these countries, with a resulting increase in the number of reports of outcomes associated with these diseases. It may be that in developing countries and, especially, in underdeveloped countries, diseases and their outcomes are mostly not reported and the real impact of some diseases may be underestimated.

In relation to the types of neurological sequelae, the most prevalent in our study was mental disability. This result was consistent with the result of the previously mentioned review and meta-analysis [7]. In both studies the mental disabilities included deficits such as delayed development, behavioral change, and intellectual deficit. There are two hypotheses for mental disabilities being the most prevalent sequelae. The first is that a change in mental state is an obligatory diagnostic criterion for defining a case of presumed encephalitis [4], so it will be a symptom present in a good portion of encephalitis patients. Another reason is the tropism of the virus for neurons of the temporal lobe and limbic system. The lesions in these regions are associated with compromised memory function, cognitive dysfunction, personality disorders, and speech disorders, among others [29], explaining the greater prevalence of sequelae related to mental disabilities.

On the other hand, the least prevalent neurological sequelae in our study was visual impairment. Despite this being scarcely mentioned in our primary studies, it is possible that this result is consistent with the literature. Due to the preference of HSV for the temporal and frontal lobes, the occipital lobe, which is jointly responsible for vision, is less affected [30]. So, visual sequelae may be less frequent. In a prospective study published in 2017 about the ophthalmologic findings in children with encephalitis, only 8% presented ophthalmologic abnormalities associated with encephalitis, with only one case (3%) caused by HSV [31].

In relation to the characteristics of our sample, the main symptoms described at the onset of encephalitis were: fever, convulsion, an altered mental state, headaches, and vomiting. An altered mental state, which is the main diagnostic criterion for defining encephalitis, was present in more than a third of the patients. Fever and convulsions, which are two lesser criteria for diagnosing probable or confirmed encephalitis [4], were present in 70% and 40% the cases, respectively. Regarding the diagnostic method for identifying the etiological agent, most of the studies carried out detection by PCR of viral DNA in the LCR, which is the gold standard for diagnosing acute viral encephalitis caused by HSV [32]. Finally, with regard to treatment, most of the studies referred to treatment with acyclovir. Two studies did not explicitly report that treatment, but the use of that medication is already well established and formally indicated in suspected encephalitis even before confirmation of the presence of the virus by PCR [2]. Therefore, we can infer that the sample included in our study corresponds to a representative portion of the children with that diagnosis and who received acyclovir treatment.

Our systematic review and meta-analysis has some limitations resulting from methodological failings and from the heterogeneity of the studies included. First, all the studies included were retrospective, which may lead to information and/or memory bias. Second, the report of the type of neurological sequelae varied between the studies; most specified the types of sequelae in a descriptive way [15–21], but two only classified the severity of the neurological sequelae presented by the patients [22, 23]. Despite these differences, we managed to categorize the sequelae presented so that we were able to analyze these data in a standardized way. Third, some information was not described in the primary studies, and despite us contacting the authors, we did not obtain any answers; this made it impossible for us to carry out other analyses and explore factors related with heterogeneity. Finally, the low quality of the studies found in our assessment tool may also be considered a limitation. We believe that this result occurs due to the fact that the tool used aimed to evaluate the strength of the causal association; as most of the studies included in our review have a cross-sectional design [15–21, 23], some items present in the tool do not apply or cannot be determined, which ultimately interferes in the final assessment of the quality of the primary studies. However, when we compared the general prevalence according to the quality of the studies, we did not find any statistically significant difference, showing that this low quality may not have interfered in the final result of our study.

We believe that this study has a number of strengths. First, our sample was representative of the general population, with diagnostic criteria, a confirmatory diagnosis, and well established treatment. In addition, this is a systematic review and meta-analysis with a greater number of patients outside the neonatal period that described the prevalence of neurological sequelae after encephalitis caused exclusively by HSV. Moreover, the search for evidence covered a considerable number of databases, with no language restrictions.

## Conclusions

Half of the children with encephalitis associated with HSV present some type of neurological sequela. Despite acyclovir treatment, half of the patients will present some type of deficiency in the follow-up; the most likely to occur is some type of mental sequela and the least likely to occur is some visual sequela. Although acute viral encephalitis has diagnostic criteria, diagnostic tests, and well established treatment, we also perceive a considerable number of patients with neurological sequelae that are scarcely explored in the studies, which usually focus on the acute phase of the infection. Quality prospective studies, with representative samples and with standardization of type and time of the outcome are needed to advance in interventions that can influence the evolution of this disease and improve the future prognosis of these children.

## Supplementary Information

**Additional file 1.** Search strategies.

## Data Availability

All data generated or analyzed during this study are included in this published article.

## Abbreviations

HSV: Herpes simplex virus
HSE: Herpes simplex encephalitis
PRISMA: Preferred Reporting Items for Systematic Reviews and Meta-Analyses
MEDLINE: Medical Literature Analysis and Retrieval System Online
EMBASE: Excerpta Medica Database
SCIELO: Scientific Electronic Library Online
LILACS: Latin American Caribbean Health Sciences Literature
CCTR: Cochrane Central Register of Controlled Trials
CINAHL: Cumulative Index to Nursing and Allied Health Literature
PSYCINFO: American Psychological Association PsycInfo
CI: Confidence interval
PCR: Polymerase chain reaction
WBC: White blood cells
IgM: Immunoglobulin M
IgG: Immunoglobulin G
CMV: Cytomegalovirus
EBV: Epstein-Barr virus
CNS: Central nervous system
CSF: Cerebrospinal fluid
EEG: Electroencephalogram
MR: Nuclear Magnetic Resonance
CT: Computed Tomography
PRISMA: Preferred Reporting Items for Systematic Reviews and Meta-Analyses
PROSPERO: International prospective register of systematic reviews
NIH: National Institute of Health Study Quality Assessment Tools
HDI: Human Development Index
DNA: Deoxyribonucleic acid
RNA: Ribonucleic acid
Mesh: Medical Subject Heading
WHO: World Health Organization
