# Supplementary material for: Neurological sequelae after encephalitis associated with herpes simplex virus in children: systematic review and meta-analysis
Source: BMC Infect Dis. 2023 Jan 26;23:55. doi: 10.1186/s12879-023-08007-3 (PMC9878875; doi:10.1186/s12879-023-08007-3)
Supplement: Supplementary file 1 — Additional file 1. Search strategies. [file 12879_2023_8007_MOESM1_ESM.docx]

**Additional file**

**Search strategies**

| **Database** | **Search strategies** |
| --- | --- |
| **MEDLINE** | #1 (child) OR (children) OR (Child Preschool) OR (Children Preschool) OR (Adolescent) OR (Adolescents) OR (Adolescence) OR (Teens) OR (Teen) OR (Teenagers) OR (Teenager) OR (Youth) OR (Youths)  #2 (Encephalitis) OR (Brain Inflammation) OR (Brain Inflammations)  #3 (Encephalitis Herpes Simplex) OR (Herpes Simplex Encephalitides) OR (Herpes Encephalitis) OR (Herpetic Encephalitis) OR (Encephalitides Herpetic) OR (Meningoencephalitis Herpes Simplex Virus) OR (Herpes Simplex Meningoencephalitis) OR (Herpetic Meningoencephalitis) OR (Herpetic Acute Necrotizing Encephalitis) OR (Simplexvirus) OR (Simplex viruses) OR (Herpes Simplex Virus) OR (Herpes Simplex Viruses) OR (HSV)  #4 (complications) OR (sequels) OR (sequelae) OR (outcome)  #5 (Neurologic Manifestations) OR (Manifestation Neurologic) OR (Neurological Manifestations) OR (Neurologic Signs and Symptoms) OR (Manifestation Neurological) OR (Neurologic Deficits) OR (Deficit Neurologic) OR (Neurologic Symptoms) OR (Neurologic Symptom) OR (Neurologic Findings) OR (Finding Neurologic) OR (Neurologic Signs) OR (Neurologic Sign) OR (Focal Neurologic Deficits) OR (Deficit Focal Neurologic) OR (Neurologic Dysfunction) OR (Dysfunctions Neurologic)  #1 AND #2 AND #3 AND #4 AND #5  (((((child) OR (children) OR (Child Preschool) OR (Children Preschool) OR (Adolescent) OR (Adolescents) OR (Adolescence) OR (Teens) OR (Teen) OR (Teenagers) OR (Teenager) OR (Youth) OR (Youths)) AND ((Encephalitis) OR (Brain Inflammation) OR (Brain Inflammations))) AND ((Encephalitis Herpes Simplex) OR (Herpes Simplex Encephalitides) OR (Herpes Encephalitis) OR (Herpetic Encephalitis) OR (Encephalitides Herpetic) OR (Meningoencephalitis Herpes Simplex Virus) OR (Herpes Simplex Meningoencephalitis) OR (Herpetic Meningoencephalitis) OR (Herpetic Acute Necrotizing Encephalitis) OR (Simplexvirus) OR (Simplex viruses) OR (Herpes Simplex Virus) OR (Herpes Simplex Viruses) OR (HSV))) AND ((complications) OR (sequels) OR (sequelae) OR (outcome))) AND ((Neurologic Manifestations) OR (Manifestation Neurologic) OR (Neurological Manifestations) OR (Neurologic Signs and Symptoms) OR (Manifestation Neurological) OR (Neurologic Deficits) OR (Deficit Neurologic) OR (Neurologic Symptoms) OR (Neurologic Symptom) OR (Neurologic Findings) OR (Finding Neurologic) OR (Neurologic Signs) OR (Neurologic Sign) OR (Focal Neurologic Deficits) OR (Deficit Focal Neurologic) OR (Neurologic Dysfunction) OR (Dysfunctions Neurologic)) |
| **EMBASE** | #1 CHILD OR PRESCHOOL OR ADOLESCENT  ('child'/exp OR 'child' OR 'children' OR 'preschool child'/exp OR 'child, preschool' OR 'pre-school child' OR 'pre-schooler' OR 'pre-schoolers' OR 'preschool child' OR 'preschooler' OR 'adolescent'/exp OR 'adolescent' OR 'teenager')  #2 ENCEPHALITIS AND HERPES SIMPLEX VIRUS OR HERPES SIMPLEX ENCEPHALITIS  ('encephalitis'/exp AND ('herpes simplex virus'/exp OR 'herpes simplex virus' OR 'hsv' OR 'human herpes virus' OR 'virus, herpes simplex') OR 'herpes simplex encephalitis'/exp OR 'hsv encephalitis' OR 'herpes simplex virus encephalitis' OR 'encephalitis, herpes simplex' OR 'encephalopathy, herpes simplex' OR 'herpes encephalitis' OR 'herpes simplex encephalitis' OR 'herpes simplex encephalopathy' OR 'herpes simplex meningoencephalitis' OR 'herpetic encephalitis' OR 'herpetic encephalopathy' OR 'herpetic meningoencephalitis')  #3 COMPLICATION AND NEUROLOGIC DISEASE  ('complication'/exp OR 'complication' OR 'complications')  AND ('neurologic disease'/exp OR 'nervous disease' OR 'nervous disorder' OR 'nervous system disease' OR 'nervous system diseases' OR 'nervous system disorder' OR 'neural disease' OR 'neurogenic disease' OR 'neurologic complaint' OR 'neurologic disease' OR 'neurologic disorder' OR 'neurologic disturbance' OR 'neurologic dysfunction' OR 'neurologic manifestations' OR 'neurologic sign' OR 'neurologic symptom' OR 'neurologic syndrome' OR 'neurological complaint' OR 'neurological deficiency' OR 'neurological disease' OR 'neurological disorder' OR 'neurological disturbance' OR 'neurological sign' OR 'neurological symptom' OR 'neurological syndrome' OR 'sign, neurologic' OR 'symptom, neurological')  #1 AND #2 AND #3  ('child'/exp OR 'child' OR 'children' OR 'preschool child'/exp OR 'child, preschool' OR 'pre-school child' OR 'pre-schooler' OR 'pre-schoolers' OR 'preschool child' OR 'preschooler' OR 'adolescent'/exp OR 'adolescent' OR 'teenager') AND ('encephalitis'/exp AND ('herpes simplex virus'/exp OR 'herpes simplex virus' OR 'hsv' OR 'human herpes virus' OR 'virus, herpes simplex') OR 'herpes simplex encephalitis'/exp OR 'hsv encephalitis' OR 'herpes simplex virus encephalitis' OR 'encephalitis, herpes simplex' OR 'encephalopathy, herpes simplex' OR 'herpes encephalitis' OR 'herpes simplex encephalitis' OR 'herpes simplex encephalopathy' OR 'herpes simplex meningoencephalitis' OR 'herpetic encephalitis' OR 'herpetic encephalopathy' OR 'herpetic meningoencephalitis') AND ('complication'/exp OR 'complication' OR 'complications' OR 'neurologic disease'/exp OR 'nervous disease' OR 'nervous disorder' OR 'nervous system disease' OR 'nervous system diseases' OR 'nervous system disorder' OR 'neural disease' OR 'neurogenic disease' OR 'neurologic complaint' OR 'neurologic disease' OR 'neurologic disorder' OR 'neurologic disturbance' OR 'neurologic dysfunction' OR 'neurologic manifestations' OR 'neurologic sign' OR 'neurologic symptom' OR 'neurologic syndrome' OR 'neurological complaint' OR 'neurological deficiency' OR 'neurological disease' OR 'neurological disorder' OR 'neurological disturbance' OR 'neurological sign' OR 'neurological symptom' OR 'neurological syndrome' OR 'sign, neurologic' OR 'symptom, neurological') |
| **COCHRANE** | child OR children OR Child Preschool OR Children Preschool OR Adolescent OR Adolescents OR Adolescence OR Teens OR Teen OR Teenagers OR Teenager OR Youth OR Youths  AND Encephalitis OR Brain Inflammation OR Brain  AND Encephalitis Herpes Simplex OR Herpes Simplex Encephalitides OR Herpes Encephalitis OR Herpetic Encephalitis OR Encephalitides Herpetic OR Meningoencephalitis Herpes Simplex Virus OR Herpes Simplex Meningoencephalitis OR Herpetic Meningoencephalitis OR Herpetic Acute Necrotizing Encephalitis OR Simplexvirus OR Simplex viruses OR Herpes Simplex Virus OR Herpes Simplex Viruses OR HSV  AND complications OR sequels OR sequelae OR outcome  AND Neurologic Manifestations OR Manifestation Neurologic OR Neurological Manifestations OR Neurologic Signs and Symptoms OR Manifestation Neurological OR Neurologic Deficits OR Deficit Neurologic OR Neurologic Symptoms OR Neurologic Symptom OR Neurologic Findings OR Finding Neurologic OR Neurologic Signs OR Neurologic Sign OR Focal Neurologic Deficits OR Deficit Focal Neurologic OR Neurologic Dysfunction OR Dysfunctions Neurologic in Title Abstract Keyword |
| **SCIELO** | ((child) OR (children) OR (preschool child) OR (preschool children) OR (adolescent) OR (adolescentes) OR (teen) OR (teens) OR (teenager) OR (teenagers) OR (youth) OR (youths))  AND ((encephalitis) OR (brain inflammation) OR (brain inflammations))  AND ((Encephalitis Herpes Simplex) OR (Encephalitides Herpes Simplex) OR (Herpes Encephalitis) OR (Herpetic Encephalitis) OR (Herpetic Encephalitides) OR (Meningoencephalitis, Herpes Simplex Virus) OR (Herpes Simplex Meningoencephalitides) OR (Meningoencephalitis Herpes Simplex) OR (Herpetic Meningoencephalitis) OR (Herpetic Meningoencephalitides) OR (Herpetic Acute Necrotizing Encephalitis) ) OR ((Simplexvirus) OR (Simplexviruses) OR (Herpes Simplex Virus) OR (Herpes Simplex Viruses) OR (HSV) OR (Encephalitides Herpes Simplex))  AND ((complications) OR (sequelae) OR (sequels) OR (outcome)) AND ((neurologic manifestations) OR (neurologic manifestation) OR (Neurological Manifestations) OR (Neurologic Signs and Symptoms) OR (Neurological Manifestation) OR (Neurologic Deficits) OR (Neurologic Deficit) OR (Neurologic Symptoms) OR (Neurologic Symptom) OR (Neurologic Findings) OR (Neurologic Finding) OR (Neurologic Signs) OR (Neurologic Sign) OR (Focal Neurologic Deficits) OR (Focal Neurologic Deficit) OR (Neurologic Dysfunction) OR (Neurologic Dysfunctions)) |
| **LILACS** | #1 (child) OR (children) OR (Child Preschool) OR (Children Preschool) OR (Adolescent) OR (Adolescents) OR (Adolescence) OR (Teens) OR (Teen) OR (Teenagers) OR (Teenager) OR (Youth) OR (Youths)  #2 (Encephalitis) OR (Brain Inflammation) OR (Brain Inflammations) AND (Encephalitis Herpes Simplex) OR (Herpes Simplex Encephalitides) OR (Herpes Encephalitis) OR (Herpetic Encephalitis) OR (Encephalitides Herpetic) OR (Meningoencephalitis Herpes Simplex Virus) OR (Herpes Simplex Meningoencephalitis) OR (Herpetic Meningoencephalitis) OR (Herpetic Acute Necrotizing Encephalitis) OR (Simplexvirus) OR (Simplex viruses) OR (Herpes Simplex Virus) OR (Herpes Simplex Viruses) OR (HSV)  #3 (complications) OR (sequels) OR (sequelae) OR (outcome) AND (Neurologic Manifestations) OR (Manifestation Neurologic) OR (Neurological Manifestations) OR (Neurologic Signs and Symptoms) OR (Manifestation Neurological) OR (Neurologic Deficits) OR (Deficit Neurologic) OR (Neurologic Symptoms) OR (Neurologic Symptom) OR (Neurologic Findings) OR (Finding Neurologic) OR (Neurologic Signs) OR (Neurologic Sign) OR (Focal Neurologic Deficits) OR (Deficit Focal Neurologic) OR (Neurologic Dysfunction) OR (Dysfunctions Neurologic)  #1 AND #2 AND #3  (child) OR (children) OR (Child Preschool) OR (Children Preschool) OR (Adolescent) OR (Adolescents) OR (Adolescence) OR (Teens) OR (Teen) OR (Teenagers) OR (Teenager) OR (Youth) OR (Youths) and (Encephalitis) OR (Brain Inflammation) OR (Brain Inflammations) AND (Encephalitis Herpes Simplex) OR (Herpes Simplex Encephalitides) OR (Herpes Encephalitis) OR (Herpetic Encephalitis) OR (Encephalitides Herpetic) OR (Meningoencephalitis Herpes Simplex Virus) OR (Herpes Simplex Meningoencephalitis) OR (Herpetic Meningoencephalitis) OR (Herpetic Acute Necrotizing Encephalitis) OR (Simplexvirus) OR (Simplex viruses) OR (Herpes Simplex Virus) OR (Herpes Simplex Viruses) OR (HSV) and (complications) OR (sequels) OR (sequelae) OR (outcome) AND (Neurologic Manifestations) OR (Manifestation Neurologic) OR (Neurological Manifestations) OR (Neurologic Signs and Symptoms) OR (Manifestation Neurological) OR (Neurologic Deficits) OR (Deficit Neurologic) OR (Neurologic Symptoms) OR (Neurologic Symptom) OR (Neurologic Findings) OR (Finding Neurologic) OR (Neurologic Signs) OR (Neurologic Sign) OR (Focal Neurologic Deficits) OR (Deficit Focal Neurologic) OR (Neurologic Dysfunction) OR (Dysfunctions Neurologic) |
| **CINAHL** | #1 child OR children OR preschool children OR preschool child OR adolescents OR teens OR teen OR teenagers OR teenager OR youth OR youths  #2 encephalitis OR brain inflammation  #3 encefalitis herpetica OR herpes simplex encephalitis OR herpes encephalitis OR meningoencephalitis OR herpes simplex virus OR herpes simplex  #4 complications OR sequelae OR sequels OR sequela OR sequelas OR outcomes  #5 neurologic manifestations OR neurological disorders OR neurological disease OR neurological outcome OR neurological signs OR neurological symptoms OR neurological deficit OR neurological findings  #1 AND #2 AND #3 AND #4 AND #5  ( child OR children OR preschool children OR preschool child OR adolescents OR teens OR teen OR teenagers OR teenager OR youth OR youths ) AND ( encephalitis OR brain inflammation ) AND ( complications OR sequelae OR sequels OR sequela OR sequelas OR outcomes ) AND ( neurologic manifestations OR neurological disorders OR neurological disease OR neurological outcome OR neurological signs OR neurological symptoms OR neurological deficit OR neurological findings ) |
| **Web of Science** | #1 TS=(child OR children OR Child Preschool OR Children Preschool OR Adolescent OR Adolescents OR Adolescence OR Teens OR Teen OR Teenagers OR Teenager OR Youth OR Youths)  #2 TS=(Encephalitis OR Brain Inflammation OR Brain Inflammations)  #3 TS=(Encephalitis Herpes Simplex OR Herpes Simplex Encephalitides OR Herpes Encephalitis OR Herpetic Encephalitis OR Encephalitides Herpetic OR Meningoencephalitis Herpes Simplex Virus OR Herpes Simplex Meningoencephalitis OR Herpetic Meningoencephalitis OR Herpetic Acute Necrotizing Encephalitis OR Simplexvirus OR Simplex viruses OR Herpes Simplex Virus OR Herpes Simplex Viruses OR HSV)  #4 TS=(complications OR sequels OR sequelae OR outcome)  #5 TS=(Neurologic Manifestations OR Manifestation Neurologic OR Neurological Manifestations OR Neurologic Signs and Symptoms OR Manifestation Neurological OR Neurologic Deficits OR Deficit Neurologic OR Neurologic Symptoms OR Neurologic Symptom OR Neurologic Findings OR Finding Neurologic OR Neurologic Signs OR Neurologic Sign OR Focal Neurologic Deficits OR Deficit Focal Neurologic OR Neurologic Dysfunction OR Dysfunctions Neurologic)  #1 AND #2 #3 AND #4 AND #5  TS=child OR children OR Child Preschool OR Children Preschool OR Adolescent OR Adolescents OR Adolescence OR Teens OR Teen OR Teenagers OR Teenager OR Youth OR Youths  and Encephalitis OR Brain Inflammation OR Brain Inflammations and Encephalitis Herpes Simplex OR Herpes Simplex Encephalitides OR Herpes Encephalitis OR Herpetic Encephalitis OR Encephalitides Herpetic OR Meningoencephalitis Herpes Simplex Virus OR Herpes Simplex Meningoencephalitis OR Herpetic Meningoencephalitis OR Herpetic Acute Necrotizing Encephalitis OR simplexviruses OR Simplex viruses OR Herpes Simplex Virus OR Herpes Simplex Viruses OR HSV and complications OR sequels OR sequelae OR outcome and Neurologic Manifestations OR Manifestation Neurologic OR Neurological Manifestations OR Neurologic Signs and Symptoms OR Manifestation Neurological OR Neurologic Deficits OR Deficit Neurologic OR Neurologic Symptoms OR Neurologic Symptom OR Neurologic Findings OR Finding Neurologic OR Neurologic Signs OR Neurologic Sign OR Focal Neurologic Deficits OR Deficit Focal Neurologic OR Neurologic Dysfunction OR Dysfunctions Neurologic |
| **PsycINFO** | #1 “child” OR “children” OR “Child Preschool” OR “Children Preschool” OR “Adolescent” OR “Adolescents” OR “Adolescence” OR “Teens” OR “Teen” OR “Teenagers” OR “Teenager” OR “Youth” OR “Youths”  #2 “Encephalitis” OR “Brain Inflammation” OR “Brain Inflammations”  #3 “Encephalitis Herpes Simplex” OR “Herpes Simplex Encephalitides” OR “Herpes Encephalitis” OR “Herpetic Encephalitis” OR “Encephalitides Herpetic” OR “Meningoencephalitis Herpes Simplex Virus” OR “Herpes Simplex Meningoencephalitis” OR “Herpetic Meningoencephalitis” OR “Herpetic Acute Necrotizing Encephalitis” OR “Simplexvirus” OR “Simplex viruses” OR “Herpes Simplex Virus” OR “Herpes Simplex Viruses” OR “HSV”  #4 “complications” OR “sequels” OR “sequelae” OR “outcome”  #5 “Neurologic Manifestations” OR “Manifestation Neurologic” OR “Neurological Manifestations” OR “Neurologic Signs and Symptoms” OR “Manifestation Neurological” OR “Neurologic Deficits” OR “Deficit Neurologic” OR “Neurologic Symptoms” OR “Neurologic Symptom” OR “Neurologic Findings” OR “Finding Neurologic” OR “Neurologic Signs” OR “Neurologic Sign” OR “Focal Neurologic Deficits” OR “Deficit Focal Neurologic” OR “Neurologic Dysfunction” OR “Dysfunctions Neurologic”  #1 AND #2 AND #3 AND #4 AND #5  “child” OR “children” OR “Child Preschool” OR “Children Preschool” OR “Adolescent” OR “Adolescents” OR “Adolescence” OR “Teens” OR “Teen” OR “Teenagers” OR “Teenager” OR “Youth” OR “Youths” AND Any Field: “Encephalitis” OR “Brain Inflammation” OR “Brain Inflammations” AND Any Field: “Encephalitis Herpes Simplex” OR “Herpes Simplex Encephalitides” OR “Herpes Encephalitis” OR “Herpetic Encephalitis” OR “Encephalitides Herpetic” OR “Meningoencephalitis Herpes Simplex Virus” OR “Herpes Simplex Meningoencephalitis” OR “Herpetic Meningoencephalitis” OR “Herpetic Acute Necrotizing Encephalitis” OR “Simplexvirus” OR “Simplex viruses” OR “Herpes Simplex Virus” OR “Herpes Simplex Viruses” OR “HSV” AND Any Field: “complications” OR “sequels” OR “sequelae” OR “outcome” AND Any Field: “Neurologic Manifestations” OR “Manifestation Neurologic” OR “Neurological Manifestations” OR “Neurologic Signs and Symptoms” OR “Manifestation Neurological” OR “Neurologic Deficits” OR “Deficit Neurologic” OR “Neurologic Symptoms” OR “Neurologic Symptom” OR “Neurologic Findings” OR “Finding Neurologic” OR “Neurologic Signs” OR “Neurologic Sign” OR “Focal Neurologic Deficits” OR “Deficit Focal Neurologic” OR “Neurologic Dysfunction” OR “Dysfunctions Neurologic” |
